# 

**Published:** 1893-10-14

**Authors:** 


**OUR NEW OFFICES,**
428, Strand, W.C., will henceforth be the Office of this Journal.

**Notices of Births, Deaths, and Marriages** are inserted in this column at a charge of 2s. 6d. for an announcement not exceeding four lines.

### NOTICE TO CORRESPONDENTS.

All MS., Letters, Books for Review, and other matters intended for the Editor should be addressed The Editor, The Lodge, Porchester Square, London, W.
The Editor cannot undertake to return rejected MS., even when accompanied by stamped directed envelope.

**Notice to Advertisers and Subscribers.**

All Advertisements, Orders for Copies of The Hospital, and Business Communications should be addressed to The Publisher, *not to the Editor*, The Hospital, 428, Strand, W.C.
The Cost of Subscription, post free, is as follows:—Per week, 2½d.; per quarter, 2s. 6d.; per half-year, 5s.; per annum, 8s. 6d Foreign:—Per Annum, 10s. 8d.; payable, in all cases, in advance.

**"Burdett's Hospital Annual," 1893.**

Fourth year of publication. 700 pp. Boards, gilt lettering. A most complete guide to British, Colonial, Indian, and American Hospitals, Dispensaries, Nursing and Convalescent Institutions, and Asylums. Price 5s. Published by The Scientific Press (Limited), 428, Strand, W.C.

**NOTICE.—The Index for Vol. XIII. (ending March, 1893) is on sale at the Office of this Journal, price 2½d., post free.**

### Scraps and Gleanings.

The Queen has sent 40 lbs. of old linen and 290 bottles of wine for the use of the patients at Westminster Hospital.

A new medical school, open to men and women on equal footing, is to be opened shortly in connection with Tufts College at Boston.

A meeting was recently held at Horfield, a suburb of Bristol, to consider the advisability of erecting an infectious diseases hospital for that district.

The outbreak of typhoid fever at Widnes is said to be largely due to climatic influence, which has, however, been accentuated by the insufficient drainage of certain parts of the town.

The friendly societies of Wisbech have this year achieved a splendid result in doubling their collection for the North Cambridgeshire Hospital and the Hunstanton Convalescent Home.

During the months of May, June, and July, 172 patients were received at the Moseley Hall Convalescent Home for Children. The sum of £46 has been received in new subscriptions during the past quarter.

A new ward, containing 25 beds, which has been added to St. Paul's Eye and Ear Hospital, Liverpool, was recently opened by the Lord Mayor of that city. Twenty-one of the beds have been presented by subscribers.

A successful Church Parade was held by the friendly societies of Tonbridge in aid of the funds of the Tonbridge Wells General, Ear, Eye, and Homœopathic Hospitals, as also for the London Hospital for Diseases of the Skin.

Hospital Saturday was observed in Brighton the third week in September, and, at the conclusion of the counting, £1,050 was announced as having been collected for distribution among the various hospitals and dispensaries of the town.

The annual Hospital Saturday collection among workpeople in the factories and workshops of Leicester was regarded as fairly satisfactory this year. The Fire Brigade voluntarily undertook a street collection for the same laudable object.

The Hounslow Cottage Hospital has been reopened for outdoor patients and accident cases. A new wing has been added, the cost of which has been defrayed by a legacy, and the main building has been renovated, the total expense being £1,000.

The Metropolitan Asylums Board have approved of the purchase of the land at Shooters Hill, near the Albert Military Hospital, for the erection of the proposed fever hospital. The scheme now only awaits the sanction of the Local Government Board.

The Society 'D'utilité Publique' of Bale has decided to establish a sanatorium for the poorer classes, who are victims to pulmonary affections. The establishment will contain accommodation for 140 inmates, at the probable cost of 150,000 francs.

We understand that the Isolation Hospitals Bill has been put down for Committee on November 2nd. As the measure is favourably regarded by both sides of the House, and all amendments have been accepted, there is ground for hope that it may see an early settlement.

An objection has been made to the proposal to erect an Infectious Diseases Hospital on a platform in Exmouth Bight, at Salthouse Lake, for the reason that such a building would be almost inaccessible in stormy weather and would prove dangerous to ships at anchor.

Special efforts were made at Portsmouth this year on behalf of the Hospital Saturday Fund, and 500 boxes and 4,000 sheets were sent out by the committee, while 100 boys were employed as collectors. The fund was devoted to the Royal Portsmouth, Portsea, and Gosport Hospital.

The Town Council of Brighton has appointed a woman as inspector of nuisances in the borough. The candidate selected was trained as a nurse at St. Bartholomew's Hospital; and her duties will include visiting the houses of the poor, in which occur cases of illness among children.

A deputation from St. Austell Local Board have lately waited upon the Rural Sanitary Authority of that district in reference to the proposed co-operation in building an infectious diseases hospital for the joint use of the inhabitants residing within the area of the two authorities.

We much regret to notice that the report of the Committee of the Sheffield General Infirmary shows a deficit of £1,808, due partly to the increase of patients, but also unfortunately to the falling off of subscriptions. A special department for the treatment of skin diseases is being contemplated.

Hospital Saturday at Gainsborough, with its house to house visitations, forms a grand conclusion to the weekly collections which have been taken since the middle of May. The proceeds, which exceed those of former years, were devoted to the Lincoln County Hospital and Leeds Eye and Ear Infirmary.

The work of the Fleetwood Cottage Hospital has been successfully carried on during the past year. It was gratifying to the committee that they could report at the annual meeting, lately held, that the total ordinary receipts have again this year exceeded ordinary expenses, leaving a considerable balance in hand.

The General Purposes Committee of the Bromley and Beckenham Joint Hospital Board report that they have taken into consideration the extra precautions notified from the Local Government Board with reference to cholera, and have made arrangements for the reception of seven male and seven female cases.

The total legacies and contributions up to the time of the foundation-stone laying of the Blackpool Infirmary, in August, yielded a total of £3,000 towards the contemplated cost of £4,000. A bazaar on an extensive scale lasting over a week, has recently been held in the town, with the view of raising another £1,000 towards the necessary building funds.

Much indignation has been caused at Stewarton (Kilmarnock) at the appearance of a hospital tent in a neighbouring field. A meeting was called to protest against the existence of the same, at which it was explained by the commissioners that a favourable site failing to be forthcoming for an isolation hospital, this temporary substitute had been provided.

### FIRST IMPRESSIONS OF BOOKS RECEIVED.

A Text Book of General Therapeutics. By W. Hale White, R.C.P. (London: Macmillan and Co.)

This book of 352 pages, crown 8vo., contains a wealth of information, including all the most recent developments. From medical climatology to hypnotism is a long journey, but the reader will find his path made easy and pleasant, and cannot fail to profit much, though he travels fast. This book is welcome and timely. It has been written by a master hand, and will no doubt have a wide popularity.

### MEDICAL VACANCIES AND APPOINTMENTS.

### APPOINTMENTS.

H. F. Burns, appointed Medical Officer for the Dent District of the Sedbergh Union. F. W. Clark, M.B., D.P.H.Camb., M.R.C.S., L.R.C.P.Lond., Assistant Medical Officer of Health to the River Tyne Port Sanitary Authority, appointed Medical Officer of Health to the Port and Borough of Lowestoft, and Medical Superintendent of the Sanatorium. E. J. Cross, M.B., C.M.Edin., appointed Medical Officer for the Southwick District of the Fareham Union. H. M. Cullinan, L. and L.M.R.C.S.I. and R.C.P.I., L.M., Rotunda Hospital, Dublin, appointed Second Assistant Medical Officer, Richmond Lunatic Asylum. F. W. H. L. Day, L.R.C.P.Lond., M.R.C.S.Eng., appointed Medical Officer of Health for Hitchin. P. V. Dodd, B.A.Dub., M.R.C.S., L.R.C.P.Lond., appointed Temporary Assistant Medical Officer of Health to the Folkestone Town Council. J. O'Connor Donelan, L. and L.M.R.C.S.I. and R.C.P.I., M.P.C., appointed First Assistant Medical Officer in the Richmond District Lunatic Asylum, Dublin. Dr. Flood, appointed Surgeon to the York Amalgamated Friendly Societies' Medical Association. D. Alex. Fraser, M.D.Brux., M.R.C.S.Eng., reappointed Medical Officer of Health for Totnes Borough. Dr. Fraser, appointed Medical Officer of Health for the Carnarvonshire Combined Sanitary District. C. Alex. Hill, B.A.Cantab., M.R.C.S.Eng., L.R.C.P. Lond., appointed Assistant House-Physician at St. George's Hospital. G. H. Hooper, M.R.C.S., L.R.C.P., appointed Resident Obstetrical Officer at Charing Cross Hospital. C. G. Jones, L.R.C.P.Edin., M.R.C.S.Eng., appointed Medical Officer of Health to the Cardigan Rural Sanitary Authority. J. C. Leach, M.D.Durh., M.R.C.S.Eng., D.P.H.Camb., appointed Coroner for the Northern District of Dorset. J. G. Macaskie, L.R.C.P., L.R.C.S.Edin., D.P.H., reappointed Medical Officer of Health to the Belford Rural Sanitary Authority. J. D. McFeeley, L.R.C.P., L.R.C.S.I., appointed *pro tem.* Medical Officer for the Kinlough Dispensary District of the Ballyshannon Union. A. J. Moore, M.R.C.S.Eng., L.S.A., reappointed Medical Officer of Health for the St. Lawrence District of the Reading Union. H. C. Nance, F.R.C.S.Eng., L.R.C.P.Lond., appointed Assistant Surgeon to the Jenny Lind Infirmary for Sick Children, Norwich. Dr. Neale, appointed Medical Officer to the Barry Port Sanitary Authority. T. Newby, M.D.St.And., M.R.C.S. Eng., L.S.A., reappointed temporary Medical Officer of Health to the Great Grimsby Town Council. P. M. O'Brien, appointed Medical Officer for the Twelvth District of the North Bierley Union. R. J. Orford, L.R.C.P.Lond., M.R.C.S.Eng., appointed Medical Officer for Market Bosworth. C. H. W. Parkinson, M.R.C.S.Eng., D.P.H.R.C.S.Edin., appointed Coroner for the Eastern District of Dorset. F. W. Pearce, M.R.C.S., L.R.C.P., appointed House-Surgeon to the Northern Branch of the Brighton, Hove, and Preston Dispensary. D. F. Rambaut, M.B, B.Ch., B.A.O., M.D.Univ.Dubl., appointed Third Assistant Medical Officer and Pathologist, Richmond Lunatic Asylum, Dublin. T. Smith, B.A., M.D., B.ch., appointed Medical Superintendent of the Royal Albert Asylum for Idiots and Imbeciles of the Northern Counties, Lancaster. S. J. Stuck, M.R.C.S.Eng., L.R.C.P.Lond., L.S.A., appointed House-Surgeon and Secretary to the Royal Isle of Wight Infirmary and County Hospital, Ryde. A. Thompson, M.D.St.And., M.R.C.S.Eng., reappointed Medical Officer of Health to the Ulverstone Rural Sanitary Authority. R. Thompson, M.D., B.S.Durh., M.R.C.S.Eng., L.R.C.P.Lond., appointed Chief Medical Referee for Queensland to the Mutual Life Association of Victoria. G. F. Welsford, B.A.Camb., M.B., M.R.C.S. Eng., appointed Medical Officer for the Washfield District of the Tiverton Union. C. F. Wightman, M.R.C.S.Eng., L.R.C.P.Lond., appointed Resident House-Surgeon, Scarborough Hospital.

### VACANCIES.

The following vacancies are announced this week, the amount of salary in each case being given after the name of the institution:

**Resident Medical Officers.**

East Riding Asylum, Beverley. Assistant Medical Officer, unmarried. Salary, £100 per annum, with board, lodging, and washing. Age between 23 and 30. Applications to C. W. Hobson, Clerk to the Visiting Committee, by October 17th.

Edmonton Union. Resident Medical Officer for the Chase Farm School, Enfield. Appointment for six months. Salary at the rate of £100 per annum, with board, washing, sitting-room, and bed-room. Applications to Francis Shelton, Clerk, 808, High Road, Lower Tottenham, by October 25th.

North-West London Hospital, Kentish Town Road, N.W. Resident Medical Officer and Assistant Resident Medical Officer. Appointments for six months. Salary of £50 attaches to the senior post. Applications to Alfred Craske, Secretary, by October 20th.

### The Hospital Advertiser.

### MEDICAL APPOINTMENTS.

THE HOSPITAL FOR SICK CHILDREN, GREAT ORMOND STREET, W.C.

SOUTH DEVON AND EAST CORNWALL HOSPITAL, PLYMOUTH.

6, Princess Square, Plymouth,

Richmond District Asylum. Resident Clinical Assistant, doubly qualified. Appointment for one year. Applications to the Resident Medical Superintendent, Richmond Asylum, Grangegoram, by October 17th.

Rotherham Hospital and Dispensary. Assistant House Surgeon. Rooms, commons, and washing provided. Appointment for six months. Applications to the House Surgeon by October 19th.

Royal Hospital for Diseases of the Chest, City Road, E.C. Resident Medical Officer. Appointment for six months, when re-election is required. Salary at the rate of £100 per annum, with furnished apartments and board. Applications to the Secretary by October 16th.

South Devon and East Cornwall Hospital, Plymouth. House Surgeon and Assistant House Surgeon. Sa ary for the House Surgeon, £100 per annum, with board and residence; for the Assistant House Surgeon (whose appointment is for six months), £10, with board and residence. Applications to J. W. Wilson, Honorary Secretary, 6, Princess Square, Plymouth, by October 18th.

Weston-Super-Mare Hospital. Medical Officer. doubly qualified. Salary, £80 per annum, with board, lodging, and washing. Applications to the Honorary Secretary by October 18th.

### Non-Resident Medical Officers.

Hospital for Sick Children, Great Ormond Street, W.C. Surgical Registrar and Anæsthetist. Appointment for one year, an honorarium of £40 being voted at the expiration of that term. Applications to the Secretary by October 24th.

South Devon and East Cornwall Hospital, Plymouth. Physician. Applications to J. W. Wilson, Honorary Secretary, 6, Princess Square, Plymouth, by October 18th.

Stratton Union, Cornwall. Medical Officer for the South District. Salary, £52 per annum, with certain fees. Applications to William Rowe, Clerk to the Guardians, Stratton, Cornwall, by October 9th.

Taunton and Somerset Hospital. Honorary Physician. Applications to J. H. Biddulph Pinchard, Secretary, 13, Hammet Street, Taunton, by October 23rd.

### PASS LISTS.

### University of London (Intermediate Science and Preliminary Scientific (M.B.) conjointly.)

Inorganic Chemistry.—*First Class:* E. J. Russell, Int.Sc. (exhibition), University College, Aberystwith; F. H. Painter, Int.Sc., private study; A. C. Wright, Int.Sc., Magdalen College, Oxford; W. H. Sodeau, Prel.Sci., King's College. *Second Class:* R. E. Barnett, Int.Sc., Royal College of Science, and private study; R. H. Pickard, Int.Sc., Mason College; A. L. White, Int.Sc., Yorkshire College, and private study; O. F. F. Grünbaum, Prel.Sci., Trinity College, Cambridge; C. Nathan, Prel.Sci, private study, and University Tutorial and Finsbury Technical Colleges. *Third Class:* W. Cammack, Int.Sc., University College, Aberystwith; W. Cormack, Int.Sc., private study; T. A. Mayo, Prel.Sci., Epsom College; E. Stenhouse, Int.Sc., Royal College of Science; A. S. Barnes, Prel.Sci., Mason College; J. G. Wells, Int.Sc., private study; T. J. Thomas, Int.Sc., University College, Aberystwith; E. A. Tyler, Int.Sc., Mansfield Grammar School and University College, Nottingham.

Experimental Physics.—*First Class:* J. W. Pickles, Int.Sc. (Neil Arnott exhibition and medal), Royal College Science, Owens College, and private study; W. H. Sodeau, Prel.Sci. (Neil Arnott medal), King's College; B. Hopkinson, Int.Sc., Trinity College, Cambridge; E. A. Tyler, Int.Sc., Mansfield Grammar School and University College, Nottingham. *Second Class:* H. T. George, Int.Sc., University College, Cardiff. *Third Class:* O. F. F. Grünbaum, Prel.Sci., Trinity College, Cambridge; A. G. Haynes, B.A., Int.Sc., Birkbeck Institution and University Tutorial and Carlyon Colleges; A. M. Stewart, Int.Sc., University College, Bangor; G. Finch, Int.Sc., University College.

Botany.—*First Class:* J. Pelling, Prel.Sc., Mason College, *Second Class:* J. G. Wells, Int.Sc., private study; F. Douglass, Int.Sc., private study. *Third Class:* E. A. N. Twigg, Int.Sc., Mason and University Tutorial Colleges; G. W. Roome, Int.Sc., University College, Bangor, and private study; F. Escombe, Int.Sc., King's College; F. J. A. Beringer, Int.Sc., private study and Tutorial College.

Zoology.—*First Class:* J. Belling, Prel. Sci. (disqualified by age for the exhibition), Mason College; A. Edmunds, Int.Sc. (exhibition), Birkbeck Institute and Islington High School. *Second Class:* H. A. Colwell, Prel.Sci., St. Bartholomew's Hospital. *Third Class:* E. A. N. Twigg, Int.Sc., Mason and University Tutorial Colleges; G. P. Tayler, Prel. Sci., St. Bartholomew's Hospital; S. A. Millen, Prel.Sci., St. Bartholomew's Hospital.

### THE EDINBURGH TRIPLE QUALIFICATION.

### Papers Recently Set.

FIRST EXAMINATION.

Questions in Physics (all of which are to be answered, and calculations shown where required).

1. Give Newton's "Law of Gravitation." Suppose a body to weigh 1 lb. at the earth's surface, what would it weigh at the height of 8,000 miles, assuming the radius of the earth to be 4,000 miles?
2. Give the rule for finding the pressure of water on the side of a cylindrical vessel containing it.
3. Explain by aid of a sketch the action of the ordinary air-pump. What appendage may be used to indicate the amount of rarefaction?
4. Describe the construction of Volta's Pile, and explain its action.

Questions in Elementary Anatomy (all of which are to be answered).

1. Describe the temporo-maxillary joint in regard to (1) the articular ends of the bones; (2) the ligaments; and (3) the movements.
2. Describe the muscles which *pronate* the radius in regard to their origin, insertion, relations, actions, nerve supply, and blood supply.
3. Describe the internal or long, and external or short, saphenous nerves.
4. Name the branches of the brachial artery in their order.

